# Demographic variation in incidence of adult glioma by subtype, United States, 1992-2007

**DOI:** 10.1186/1471-2407-11-325

**Published:** 2011-07-29

**Authors:** Robert Dubrow, Amy S Darefsky

**Affiliations:** 1 Yale School of Public Health, Yale School of Medicine, P.O. Box 208034, New Haven, CT 06520-8034, USA

## Abstract

**Background:**

We hypothesized that race/ethnic group, sex, age, and/or calendar period variation in adult glioma incidence differs between the two broad subtypes of glioblastoma (GBM) and non-GBM. Primary GBM, which constitute 90-95% of GBM, differ from non-GBM with respect to a number of molecular characteristics, providing a molecular rationale for these two broad glioma subtypes.

**Methods:**

We utilized data from the Surveillance, Epidemiology, and End Results Program for 1992-2007, ages 30-69 years. We compared 15,088 GBM cases with 9,252 non-GBM cases. We used Poisson regression to calculate adjusted rate ratios and 95% confidence intervals.

**Results:**

The GBM incidence rate increased proportionally with the 4^th ^power of age, whereas the non-GBM rate increased proportionally with the square root of age. For each subtype, compared to non-Hispanic Whites, the incidence rate among Blacks, Asians/Pacific Islanders, and American Indians/Alaskan Natives was substantially lower (one-fourth to one-half for GBM; about two-fifths for non-GBM). Secondary to this primary effect, race/ethnic group variation in incidence was significantly less for non-GBM than for GBM. For each subtype, the incidence rate was higher for males than for females, with the male/female rate ratio being significantly higher for GBM (1.6) than for non-GBM (1.4). We observed significant calendar period trends of increasing incidence for GBM and decreasing incidence for non-GBM. For the two subtypes combined, we observed a 3% decrease in incidence between 1992-1995 and 2004-2007.

**Conclusions:**

The substantial difference in age effect between GBM and non-GBM suggests a fundamental difference in the genesis of primary GBM (the driver of GBM incidence) versus non-GBM. However, the commonalities between GBM and non-GBM with respect to race/ethnic group and sex variation, more notable than the somewhat subtle, albeit statistically significant, differences, suggest that within the context of a fundamental difference, some aspects of the complex process of gliomagenesis are shared by these subtypes as well. The increasing calendar period trend of GBM incidence coupled with the decreasing trend of non-GBM incidence may at least partly be due to a secular trend in diagnostic fashion, as opposed to real changes in incidence of these subtypes.

## Background

In a previous descriptive study of international variation in the incidence of adult primary brain cancer, we found that: 1) White populations had the highest incidence rates, North American Blacks had intermediate rates, and populations of eastern and southeastern Asian origin had the lowest rates; 2) incidence rates increased less steeply with age in the latter populations; 3) the male/female incidence rate ratio was between 1.4 and 1.5 among populations throughout the world; and 4) the male/female rate ratio was higher for peri- and post-menopausal ages than for pre-menopausal ages [1]. The lower incidence rate among non-Whites and the male excess had previously been noted by other investigators [2-6].

Although we examined brain cancer as a whole (due to limitations of the data we were unable to examine variation by morphologic type), our results were most likely driven by variation in the incidence of glioma, the predominant morphologic type of adult primary malignant brain tumors [2,6]. However, because gliomas themselves are heterogeneous, we now hypothesize that race/ethnic group, sex, and/or age variation in adult glioma incidence differs by morphologic subtype. To test these hypotheses, we utilized data from the population-based cancer registries of the National Cancer Institute's Surveillance, Epidemiology, and End Results (SEER) Program. We also tested the hypothesis that calendar period trends in adult glioma incidence varied by subtype.

The three main categories of adult glioma according to traditional pathological classification are astrocytoma, oligodendroglioma, and mixed oligoastrocytoma [7-9]. Each of these categories is further sub-classified by grade (grade II, grade III, or grade IV, the latter grade reserved for glioblastoma [GBM], which is grade IV astrocytoma) [8,9]. Secondary GBM develops through progression from a lower grade astrocytoma, whereas primary GBM appears to arise *de novo*, with no evidence of a lower-grade precursor [9].

There is considerable variability in the diagnosis of the traditional glioma categories and grades among pathologists, across geographic regions, and over time [10]. Nevertheless, epidemiologists and neuropathologists have reached a consensus that a pathology report of GBM is likely to be valid (although a small proportion of GBMs may be incorrectly classified as grade III [anaplastic] astrocytomas), whereas valid classification of non-GBM subtypes requires centralized pathologic review [10]. Thus, adult gliomas can validly be categorized broadly as GBM versus non-GBM based on pathology report diagnoses (which are utilized by SEER cancer registries), but non-GBM cannot be sub-divided further without compromising validity. Primary GBM, which constitute 90-95% of GBM [9], differ from non-GBM with respect to DNA copy number [11], gene expression [11], DNA methylation [12-14], and isocitrate dehydrogenase (IDH) 1 or 2 gene mutation status [11-16], providing a molecular rationale for the two broad glioma subtypes of GBM and non-GBM.

## Methods

### Study population

We utilized publicly-available data from the SEER population-based cancer registries (SEER Research Data [1973-2007], released April 2010, based on the November 2009 submission) and associated SEER U.S. population data (SEER Program Populations [1969-2007] http://www.seer.cancer.gov/popdata, released November 2009) [17]. We included in our analyses adult glioma cases diagnosed between 1992 and 2007. Cases diagnosed between 1992 and 1999 were from the SEER 13 Registries Database (Alaska Native, Atlanta, Connecticut, Detroit, Hawaii, Iowa, Los Angeles, New Mexico, Rural Georgia, San Francisco-Oakland, San Jose-Monterey, Seattle-Puget Sound, and Utah). Cases diagnosed between 2000 and 2007 were from the SEER 17 Registries Database (SEER 13 plus Greater California, Kentucky, Louisiana, and New Jersey). The SEER 13 registries cover about 14% and the SEER 17 registries cover about 26% of the total U.S. population.

We restricted our analyses to adult glioma because different morphologic types of glioma predominate in adults compared to children [2,5,6]. We defined glioma as a malignant brain neoplasm (International Classification of Diseases for Oncology, third edition [ICD-O-3] topography codes C710-C719 and behavior code 3) with an ICD-O-3 morphology code of 9380 (glioma, malignant; glioma, not otherwise specified [NOS]), 9382 (mixed glioma), 9400 (astrocytoma, NOS), 9401 (astrocytoma, anaplastic), 9410 (protoplasmic astrocytoma), 9411 (gemistocytic astrocytoma), 9420 (fibrillary astrocytoma), 9440 (glioblastoma, NOS), 9441 (giant cell glioblastoma), 9442 (gliosarcoma), 9450 (oligodendroglioma, NOS), 9451 (oligodendroglioma, anaplastic), or 9460 (oligodendroblastoma) [18]. We defined GBM as ICD-O-3 morphology codes 9440, 9441, and 9442. We defined non-GBM as all other codes except 9380 (glioma, malignant; glioma, NOS).

For valid classification of adult glioma as GBM versus non-GBM, we made two necessary exclusions. First, we excluded cases with the non-specific code 9380 because such cases could not be classified as GBM versus non-GBM. Second, we excluded cases that were not microscopically confirmed because valid distinction between GBM and non-GBM requires microscopic examination of tumor tissue and cannot be accomplished by imaging alone [19-21].

Given these exclusions, we sought to minimize selection bias due to differences in the proportion of cases excluded according to age, calendar period, sex, or race/ethnic group. First, we required that the proportion of cases that were both microscopically confirmed and had a specific morphology code (a code other than 9380) be greater than 90% in each included 5-year age group, resulting in restriction of the age range to 30-69 years (Table 1). Overall, in this age range 92.3% of cases were both microscopically confirmed and had a specific morphology code. Then, we examined this proportion within this age range according to period, sex, and race/ethnic group and found that the proportion did not vary meaningfully by these characteristics, although it was slightly lower among Blacks (89.8%) and Asians/Pacific Islanders (89.9%) than among other race/ethnic groups (Table 1). Overall, in the age range 30-69 years, 4.6% of cases were excluded due to having morphology code 9380 and 3.1% were excluded due to lack of microscopic confirmation (3.8% of GBM cases and 2.4% of non-GBM cases). We included a total of 15,088 GBM cases and 9,252 non-GBM cases in our analyses.

**Table 1:** Number and percent of glioma cases that were both microscopically confirmed and had specific ICD-O-3 morphology codes (not 9380), by demographic characteristic, SEER, 1992-2007

Characteristic	Glioma Cases		
	
	**Total**^a^	Microscopically confirmed with specific morphology	
	
	N	N	Percent
			
**Age (years)**			
20-24	812	699	86.1%
25-29	1,308	1,171	89.5%
**30-34**	**1,709**	**1,553**	**90.9%**
**35-39**	**2,194**	**2,038**	**92.9%**
**40-44**	**2,730**	**2,522**	**92.4%**
**45-49**	**3,224**	**3,003**	**93.1%**
**50-54**	**3,879**	**3,607**	**93.0%**
**55-59**	**4,133**	**3,835**	**92.8%**
**60-64**	**4,253**	**3,917**	**92.1%**
**65-69**	**4,252**	**3,865**	**90.9%**
70-74	4,530	3,948	87.2%
75-79	4,081	3,353	82.2%
80-84	2,579	1,793	69.5%
85+	1,448	663	45.8%
			
**Total, age 30-69 years**	**26,374**	**24,340**	**92.3%**
			
**Period (age 30-69 years)**			
1992-1995	4,121	3,813	92.5%
1996-1999	4,345	4,006	92.2%
2000-2003	8,606	7,916	92.0%
2004-2007	9,302	8,605	92.5%
			
**Sex (age 30-69 years)**			
Male	15,607	14,414	92.4%
Female	10,767	9,926	92.2%
			
**Race/ethnic group (age 30-69 years)**			
Non-Hispanic White	20,860	19,354	92.8%
Hispanic White	2,735	2,487	90.9%
Black	1,444	1,297	89.8%
Asian/Pacific Islander	1,222	1,098	89.9%
American Indian/Alaskan Native			
Native	113	104	92.0%

^a^GBM (codes 9440, 9441, 9442), non-GBM (codes 9382, 9400, 9401, 9410, 9411, 9420, 9450, 9451, 9460), and non-specific glioma, malignant (9380)

### Data analysis and statistical methods

We downloaded sex-, age-, race-, Hispanic-ethnicity-, and calendar-year-specific numbers of cases and person-years at risk from the SEER website. We classified age into eight five-year age groups ranging from 30-34 to 65-69. We divided calendar-years into four four-year periods (1992-1995, 1996-1999, 2000-2003, and 2004-2007). Our race/ethnic group categories were non-Hispanic White, Hispanic White, Black, Asian/Pacific Islander, and American Indian/Alaskan Native.

For each of our two glioma subtypes, we calculated sex-and age-specific incidence rates (for each race/ethnicity group) by dividing the number of cases by the number of person-years at risk. We calculated sex-specific, age-standardized incidence rates (ASRs) and 95% confidence intervals (CIs) (for each race/ethnicity group) by direct standardization of the age-specific incidence rates to the 2000 U.S. Standard Population (Census P25-1130). We used Poisson regression to calculate adjusted rate ratios (RRs) and 95% CIs. We modeled age as a categorical variable or as the natural logarithm of age (log_e_(age)), where age was defined as the midpoint of the five-year age group. We modeled calendar period as a categorical or interval variable, the latter being used to calculate the p-value for period trend. Results for models with and without adjustment for cancer registry did not meaningfully differ; we only present the unadjusted results. To determine a p-value for heterogeneity, we entered the appropriate cross-product term into the Poisson model and conducted a likelihood ratio test for its addition, with the appropriate degrees of freedom. To further explore the relationship between glioma subtype incidence and age, we calculated adjusted RRs for 5-year age groups using Poisson regression and used the calculated RRs to perform weighted linear least squares regression of log_10_(RR) versus log_10_(age). We also performed weighted linear least squares regression of log_10_(age-specific incidence rate) versus log_10_(age). We performed Poisson regressions using Proc Genmod of SAS version 9.1; statistical tests were two-sided with α = 0.05.

## Results

### Race/ethnic group

In Table 2, we tested the hypothesis that race/ethnic group variation in incidence differs according to glioma subtype. The reference group for the RRs was non-Hispanic Whites. Except for American Indians/Alaskan Natives, 95% CIs for ASRs and RRs were relatively tight due to large numbers of cases. For each subtype, ASRs and RRs varied by race/ethnic group similarly for males and females. For GBM, rates varied about three-to-four-fold, with highest rates among non-Hispanic Whites, followed by Hispanic Whites, Blacks, Asian/Pacific Islanders, and American Indians/Alaskan Natives. As with GBM, rates for non-GBM were highest among non-Hispanic Whites, followed by Hispanic Whites, with rates among Blacks, Asian/Pacific Islanders, and American Indians/Alaskan Natives each about 40% of the rates among non-Hispanic Whites. The main differences in race/ethnic variation between GBM and non-GBM were that Blacks had lower RRs, and Asian/Pacific Islanders and American Indians/Alaskan Natives had higher RRs, for non-GBM than for GBM. Overall, race/ethnic group variation was less for non-GBM than for GBM. The difference in race/ethnic variation between GBM and non-GBM was highly significant among both males and females (p-value for heterogeneity < 0.0001 in each sex).

**Table 2:** Sex- specific, age-standardized incidence rates, age- and period-adjusted rate ratios, and age- and period-adjusted male/female rate ratios, by race/ethnic group and glioma subtype, age 30 to 69 years, SEER, 1992-2007

Glioma								
Subtype	Race/ethnic group	Males			Females			Male/Female
		
		Cases	ASR^a ^(95% CI^b^)	RR^c ^(95% CI^b^)	Cases	ASR^a ^(95% CI^b^)	RR^c ^(95% CI^b^)	RR^d ^(95% CI^b^)
							
**GBM**								
	Non-Hispanic White	7,458	5.20 (5.06, 5.32)	1.00 (reference)	4,745	3.16 (3.07, 3.25)	1.00 (reference)	1.64 (1.58, 1.70)
	Hispanic White	769	3.08 (2.86, 3.31)	0.58 (0.54, 0.62)	625	2.34 (2.16, 2.53)	0.74 (0.68, 0.80)	1.31 (1.18, 1.45)
	Black	470	2.51 (2.28, 2.74)	0.48 (0.44, 0.53)	352	1.56 (1.40, 1.73)	0.49 (0.44, 0.55)	1.61 (1.40, 1.85)
	Asian/Pacific Islander	372	1.94 (1.75, 2.14)	0.38 (0.34, 0.42)	245	1.12 (0.98, 1.26)	0.35 (0.31, 0.40)	1.74 (1.48, 2.05)
	American Indian/Alaskan Native	29	1.26 (0.79, 1.73)	0.25 (0.17, 0.36)	23	0.95 (0.56, 1.35)	0.30 (0.20, 0.45)	1.36 (0.78, 2.34)
	Total	9,098			5,990			1.61^e ^(1.56, 1.66)
							
**Non-GBM**								
	Non-Hispanic White	4,175	3.01 (2.91, 3.10)	1.00 (reference)	2,976	2.12 (2.04, 2.19)	1.00 (reference)	1.42 (1.36, 1.49)
	Hispanic White	577	1.85 (1.69, 2.00)	0.59 (0.54, 0.65)	516	1.64 (1.49, 1.78)	0.76 (0.70, 0.84)	1.10 (0.98, 1.24)
	Black	269	1.30 (1.14, 1.45)	0.43 (0.38, 0.49)	206	0.85 (0.74, 0.97)	0.40 (0.35, 0.46)	1.52 (1.26, 1.82)
	Asian/Pacific Islander	264	1.29 (1.13, 1.45)	0.43 (0.38, 0.49)	217	0.95 (0.82, 1.08)	0.45 (0.39, 0.52)	1.35 (1.13, 1.62)
	American Indian/Alaskan Native	31	1.24 (0.79, 1.69)	0.40 (0.28, 0.57)	21	0.77 (0.44, 1.11)	0.37 (0.24, 0.57)	1.54 (0.88, 2.67)
	Total	5,316			3,936			1.38^e ^(1.32, 1.44)

^a^Age-standardized rate (per 100,000 person-years; 2000 U.S. Standard Population (Census P25-1130))

^b^Confidence interval

^c^Sex-specific, race/ethnic group rate ratio, adjusted for age and period by Poisson regression

^d^Race/ethnic group-specific male/female rate ratio, adjusted for age and period by Poisson regression

^e^Male/female rate ratio, adjusted for race/ethnic group, age, and period by Poisson regression

### Sex

In Table 2, we also tested the hypothesis that sex variation in incidence differs according to glioma subtype. For each race/ethnic group and glioma subtype, males had a higher incidence rate than females. For each race/ethnic group, the male/female RR was higher for GBM than for non-GBM, except for American Indians/Alaska Natives (for whom the 95% confidence intervals were wide). The overall male/female RR, derived from a Poisson model with sex as the independent variable and age, period, and race/ethnic group as covariates, was 1.61 (95% CI = 1.56, 1.66) for GBM versus 1.38 (95% CI = 1.32, 1.44) for non-GBM. This difference was highly significant (p-value for heterogeneity < 0.0001).

The p-value for heterogeneity for the variation in male/female RR by race/ethnic group was 0.0013 for GBM and 0.0011 for non-GBM, indicating that the male/female RR varied by race/ethnic group for each glioma subtype. However, when we excluded Hispanic Whites, among whom we observed the lowest male/female RR for both GBM and non-GBM, the p-value for heterogeneity was no longer significant (p = 0.76 for GBM and p = 0.85 for non-GBM).

In Table 3 we tested the hypothesis that the male/female RR is higher for peri- and post-menopausal ages than for pre-menopausal ages. The male/female RR did not significantly vary among these menopausal age groups (p-value for heterogeneity = 0.38 for GBM and 0.087 for non-GBM). Because we observed in our previous study that the male/female RR was higher for peri- and post-menopausal ages than for pre-menopausal ages for adult brain cancer as a whole [1], most of which is glioma, in the current study we also examined GBM and non-GBM combined (data not shown) and did observe a borderline-significantly higher male/female RR for peri- and post-menopausal ages than for pre-menopausal ages (p-value for heterogeneity = 0.047).

**Table 3:** Age-, period-, and race/ethnic group-adjusted male/female rate ratios by menopausal age group and glioma subtype, age 30 to 69 years, SEER, 1992-2007

	GBM		Non-GBM	
	
Age (years)	N	RR^a ^(95% CI^b^)	N	RR^a ^(95% CI^b^)
				
30-44 (pre-menopausal)	2,056	1.70 (1.56, 1.86)	4,057	1.33 (1.25, 1.41)
45-54 (peri-menopausal)	4,135	1.61 (1.51, 1.71)	2,475	1.36 (1.25, 1.47)
55-69 (post-menopausal)	8,897	1.59 (1.52, 1.66)	2,720	1.48 (1.37, 1.60)
p-value for heterogeneity**^c^**		0.38		0.087

^a^Adjusted for age (5-year age categories), period, and race/ethnic group by Poisson regression

^b^Confidence interval

^c^Calculated from the likelihood ratio test for the addition of the cross-product term for sex by menopausal age group to the Poisson model

### Age

In Poisson models including age (categorical variable with 5-year age groups), sex, and a cross-product term between age and sex, adjusted for period and race/ethnic group, the p-value for the cross-product term was 0.37 for GBM and 0.12 for non-GBM, indicating homogeneous age effects in males and females for GBM and non-GBM, respectively. However, the difference in age effect between GBM and non-GBM was highly significant (p-value for heterogeneity < 0.0001).

**Figure 1:**
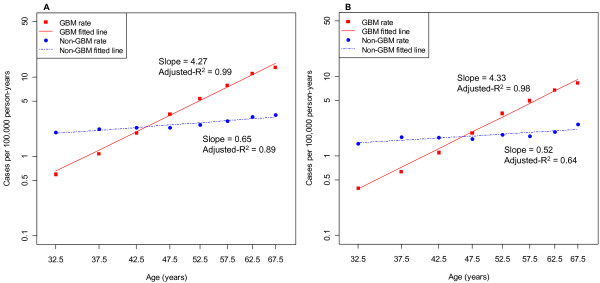
**Sex-specific log_10_-log_10 _plots of age-specific incidence rates versus age for GBM and non-GBM, respectively, age 30 to 69 years, SEER, 1992-2007**. The logarithm_10 _of the age-specific incidence rate was plotted against the logarithm_10 _of the midpoint of each 5-year age group. (A) Males; (B) Females.

To further explore the hypothesis that age variation in incidence differs according to glioma subtype, for each subtype we calculated adjusted RRs for age by 5-year age group using Poisson regression, adjusting for period, race/ethnic group, and sex. We then used these adjusted RRs in weighted least squares regressions of log_10_(RR) versus log_10_(age) and found reasonable fits, with the GBM incidence rate increasing proportionally with approximately the 4^th ^power of age (slope = 4.17; 95% CI: 3.80, 4.54; adjusted-R^2 ^= 0.99), and the non-GBM incidence rate increasing proportionally with approximately the square root of age (slope = 0.48; 95% CI = 0.25, 0.72; adjusted-R^2 ^= 0.78), showing the marked difference in age effect between GBM and non-GBM. We then calculated unadjusted RRs by 5-year age group and subtype using Poisson regression and used the unadjusted RRs in weighted least squares regression of log_10_(RR) versus log_10_(age). We found slopes and adjusted-R^2^s similar to those found when using adjusted RRs, showing that it would be valid to plot log_10_(age-specific incidence rate) versus log_10_(age) without adjustment, which we show in Figure 1.

Finally, we modeled age as log_e_(age) in Poisson models (Table 4) and found the period-, sex-, and race/ethnic-group-adjusted regression coefficients (intersections of "Total" row and "Total" column) to be identical to the corresponding slopes observed in the weighted least squares regression models that used the adjusted RRs for 5-year age groups. The p-value for heterogeneity for the sex difference in the log_e_(age) effect was 0.33 for GBM and 0.18 for non-GBM, confirming the sex homogeneity; the p-value for heterogeneity for the difference in log_e_(age) effect between GBM and non-GBM was highly significant (p < 0.0001).

**Table 4:** Race/ethnic group-specific, period-adjusted Poisson regression coefficients (slopes) for the natural logarithm of age (log_e_(age)) by glioma subtype, age 30 to 69 years, SEER, 1992-2007


**Glioma**		**Male^a^**	**Female^a^**	**Total^b^**
		
**Subtype**	**Race/ethnic group**	**Slope (log_e_(age)) (95% CI^c^)**	**Slope (log_e_(age)) (95% CI^c^)**	**Slope (log_e_(age)) (95% CI^c^)**

				
**GBM**				
	Total**^d^**	4.12 (4.01, 4.23)	4.24 (4.10, 4.38)	4.17 (4.08, 4.26)
	Non-Hispanic White	4.09 (3.96, 4.21)	4.23 (4.07, 4.39)	4.14 (4.05, 4.24)
	Hispanic White	4.51 (4.17, 4.86)	4.39 (3.99, 4.78)	4.46 (4.20, 4.72)
	Black	4.29 (3.81, 4.76)	4.38 (3.83, 4.94)	4.33 (3.97, 4.69)
	Asian/Pacific Islander	3.70 (3.19, 4.21)	3.83 (3.19, 4.47)	3.75 (3.35, 4.15)
	American Indian/Alaskan Native	3.03 (1.28, 4.77)	4.39 (2.21, 6.56)	3.59 (2.23, 4.94)
	p-value for heterogeneity^e^	0.044	0.68	0.021
				
**Non-GBM**				
	Total**^d^**	0.52 (0.40, 0.64)	0.43 (0.29, 0.56)	0.48 (0.39, 0.57)
	Non-Hispanic White	0.43 (0.29, 0.56)	0.38 (0.22, 0.54)	0.41 (0.30, 0.51)
	Hispanic White	1.13 (0.77, 1.49)	0.76 (0.38, 1.14)	0.95 (0.69, 1.21)
	Black	0.88 (0.34, 1.41)	0.68 (0.07, 1.29)	0.79 (0.39, 1.19)
	Asian/Pacific Islander	0.38 (-0.15, 0.91)	0.08 (-0.51, 0.66)	0.24 (-0.15, 0.63)
	American Indian/Alaskan Native	0.46 (-1.13, 2.05)	0.01 (-2.29, 1.61)	0.14 (-1.09, 1.38)
	p-value for heterogeneity^e^	0.0053	0.22	0.0005

^a^Log_e_(age) coefficient adjusted for period by Poisson regression

^b^Log_e_(age) coefficient adjusted for period and sex by Poisson regression

^c^Confidence interval

^d^Log_e_(age) coefficient additionally adjusted for race/ethnic group.

**^e^**Calculated from the likelihood ratio test for the addition of the cross-product term for race/ethnic group by log_e_(age) to the Poisson model

In Table 4, we also tested the hypothesis that the log_e_(age) effect differs according to race/ethnic group for each subtype. For GBM, the slope for log_e_(age) for each race/ethnic group was approximately 4. However, there was some evidence for heterogeneity (p-value for heterogeneity = 0.021), with Asians/Pacific Islanders and American Indians/Alaskan Natives having smaller slopes than the other race/ethnic groups. For non-GBM, the slopes by race/ethnic group ranged form 0.14 to 0.95, with a p-value for heterogeneity of 0.0005, with the smallest slopes again observed among Asians/Pacific Islanders and American Indians/Alaskan Natives.

### Calendar period

In Table 5 we tested the hypothesis that calendar period variation in incidence differed according to glioma subtype. For GBM, we observed a significant trend of increasing RR with more recent period; however, the magnitude of the increase in incidence rate was not large (6% increase between 1992-1995 and 2004-2007). For non-GBM, we observed a significant trend of decreasing RR with more recent period, with a 16% decrease in incidence rate between 1992-1995 and 2004-2007. The difference in period effect between GBM and non-GBM was highly significant (p-value for heterogeneity < 0.0001). Overall, for GBM and non-GBM combined, we observed a slight trend of decreasing incidence rate with more recent period (3% decrease between 1992-1995 and 2004-2007).

**Table 5:** Age-, sex-, and race/ethnic group-adjusted rate ratios for period by glioma subtype, age 30 to 69 years, SEER, 1992-2007

	GBM		Non-GBM		Total^a^	
	
Period	N	RR^b ^(95% CI^c^)	N	RR^b ^(95% CI^c^)	N	RR^b ^(95% CI^c^)
						
1992-1995	2,181	1.00 (reference)	1,632	1.00 (reference)	3,813	1.00 (reference)
1996-1999	2,351	1.03 (0.97, 1.09)	1,655	0.97 (0.91, 1.04)	4,006	1.00 (0.96, 1.05)
2000-2003	4,930	1.02 (0.97, 1.07)	2,986	0.87 (0.82, 0.92)	7,916	0.95 (0.92, 0.99)
2004-2007	5,626	1.06 (1.01, 1.12)	2,979	0.84 (0.79, 0.89)	8,605	0.97 (0.93, 1.01)
p-value, trend^d^		0.012		< 0.0001		0.037

^a^GBM and non-GBM combined

^b^Adjusted for age, sex, and race/ethnic group by Poisson regression

^c^Confidence interval

^d^Calculated from the likelihood ratio test for the addition of period (as an interval variable) to the model

## Discussion

Although we observed statistically significant differences between GBM and non-GBM in the variation of incidence rates according to age, race/ethnic group, and sex (we will discuss period separately), only the difference in age effect, which has been observed by others [2,5,22], was sizeable, with the incidence rate of GBM rising steeply with age and the incidence rate of non-GBM rising comparatively slightly with age. In spite of the statistically significant differences, the commonalities between GBM and non-GBM with respect to race/ethnic group variation and sex were more notable than the somewhat subtle differences.

### Age

The GBM incidence rate increased in proportion to approximately the 4^th ^power of age, similar to what was found in an analysis of SEER data among Whites in 1973-1982 [22]. Because secondary GBM cases are known to have a younger age distribution than primary GBM cases [9], the increase in primary GBM incidence with age (after removing secondary GBM) must be somewhat steeper, rising toward the 5^th ^power, which would make the age-incidence curve for primary GBM similar to that of many carcinomas, which typically increase in incidence approximately in proportion to between the 4^th ^and 6^th ^power of age [23]. On the other hand, the non-GBM incidence rate increased in proportion to approximately the square root of age, an age-incidence pattern that is quite unusual [23]. The previous SEER analysis among Whites in 1973-1982 found slopes of 1.7 for astrocytomas and 1.0 for oligodendrogliomas [22].

The pronounced difference in age-incidence curves between GBM (mainly primary GBM) and non-GBM suggests a fundamental difference in the genesis of these glioma subtypes, as has been suggested previously [22]. Mathematical models of carcinogenesis suggest that development of cancer types with steep log(incidence)-log(age) slopes, such as primary GBM, involve a large number of stable genetic or epigenetic changes (slope plus one; thus 5-6 changes for primary GBM) [24,25] or rapid expansion of a premalignant clone [26], whereas development of cancer types with shallow log(incidence)-log(age) slopes, such as non-GBM, involve a small number of stable genetic or epigenetic changes (as few as one or two) or slow expansion of a premalignant clone.

Indeed, GBM exhibits greater molecular complexity than non-GBM, with the implication that the path to GBM involves a greater number of molecular alterations [7]. Furthermore, the steeper age-incidence curve for GBM may indicate a greater role for age-related processes such as immunosenescence [27] or reduced efficiency of DNA repair mechanisms [28]. Given that among malignancies (brain or otherwise), non-GBM (as well as secondary GBM) are unique in exhibiting a high frequency (as high as 80%) of IDH mutations [15,16,29,30], which appear to be early events in non-GBM development [16], one can speculate that these mutations drive an atypical path to malignancy.

For both GBM and non-GBM, the log(age) slope was similar for males and females. However, within the context of the large difference in age-incidence curves between GBM and non-GBM, we observed subtle, but statistically significant, variation in age-incidence curves among race/ethnic groups. For both subtypes, Asians/Pacific Islanders and American Indians/Alaskan Natives had the smallest slopes. We observed a similar result for Asians/Pacific Islanders in our previous work on brain cancer as a whole [1].

### Race/ethnic group

For race/ethnic group variation, we observed an important commonality between GBM and non-GBM. For each subtype, compared to non-Hispanic Whites, the incidence rate among Blacks, Asian/Pacific Islanders, and American Indians/Alaskan Natives was substantially lower (one-fourth to one-half for GBM; about two-fifths for non-GBM). However, secondary to this primary effect, race/ethnic group variation in incidence was less for non-GBM than for GBM, a difference that was highly statistically significant but only moderate in magnitude.

There is evidence for race/ethnic group differences in genetic pathways to glioma [31-33]. Furthermore, genome-wide association studies have identified several genetic susceptibility regions for glioma [34,35]. Given the genotype variability across race/ethnic groups [36], it is possible that variation in the frequency of susceptibility alleles across race/ethnic groups explains at least some of the race/ethnic group variation in glioma incidence, including the race/ethnic group heterogeneity in the relationship between glioma incidence and age. The commonality between GBM and non-GBM in race/ethnic group variation suggests that at least some of the susceptibility loci that may help explain race/ethnic group variation in glioma incidence would be the same for GBM and non-GBM, although some susceptibility loci appear to show specificity with respect to glioma subtype [37-39].

### Sex

As with race/ethnic group variation, we observed an important commonality between GBM and non-GBM for sex. For each subtype, the incidence rate was higher for males than for females; this male excess of glioma is well known [2,3,5,6]. However, we did find the male/female RR to be somewhat higher for GBM (1.6) than for non-GBM (1.4), a result that was highly statistically significant. We previously suggested that the male/female difference in brain cancer incidence is biologically based [1], and that an explanation should be sought in genetic differences between males and females, sex hormones, and/or female reproductive factors [40]. Now we would add that any explanation should take into account the difference in the male/female RR between glioma subtypes.

For GBM and non-GBM, respectively, we observed similar male/female RRs among race/ethnic groups, with the exception of Hispanic Whites, among whom the male/female RR was anomalously low. Further work is required to determine whether the latter result has a biological basis or stems from an unidentified bias. It is noteworthy that the quality of the SEER Hispanic ethnicity variable was found to be moderate to substantial, but not excellent [41].

In our previous work, we found that for adult brain cancer as a whole (most of which are glioma), the male/female RR was higher in the peri- and post-menopausal age range than in the pre-menopausal age range [1]. However, in the current study, we found no evidence for an association between male/female RR and age for GBM and only suggestive evidence (p = 0.09) for a higher RR in the post-menopausal age range for non-GBM. We also found that the result for all glioma appeared to have been confounded by glioma subtype, with non-GBM both having a lower male/female RR and occurring at younger ages compared to GBM, which would explain the result from our previous work.

### Calendar period

We observed trends of increasing GBM incidence rate and decreasing non-GBM incidence rate with more recent period, with an overall trend for GBM and non-GBM combined of slightly decreasing rate. Although the latter trend was statistically significant (p = 0.037), we do not conclude that the overall incidence of glioma decreased over the 16 year period of observation because the small 3% decrease between 1992-1995 and 2004-2007 could easily be explained by an unrecognized bias. Furthermore, given the well-known inter-observer variability in glioma diagnosis [10,42-45], the increasing trend of GBM incidence coupled with the decreasing trend of non-GBM incidence may at least partly be due to a secular trend in diagnostic fashion, as opposed to real changes in incidence of these two subtypes.

### Limitations

Due to the known inter-observer variability in the detailed morphologic classification of non-GBM in particular [10], we did not attempt to sub-classify these tumors into the traditional categories of astrocytoma, oligodendroglioma, and mixed oligoastrocytoma or by grade. Furthermore, we were unable to distinguish primary from secondary GBM and were unable to take into account known molecular heterogeneity within the two broad categories of GBM [46] and non-GBM [47]. Another limitation was possible selection bias due to differential diagnosis of microscopically confirmed glioma that had a specific morphology code (not the nonspecific code 9380), according to demographic factors. However, our restriction to ages 30-69 years, an age range with a high proportion of cases both microscopically confirmed and with a specific morphology code, tended to minimize the potential for any such bias appreciably distorting the results. This restriction was at the expense of generalizability to a broader age range.

## Conclusions

We found statistically significant differences between GBM and non-GBM in the variation of incidence rates according to age, race/ethnic group, sex, and period. The difference in age effect, which was substantial, suggests a fundamental difference in the genesis of primary GBM (the driver of GBM incidence) versus non-GBM. However, the commonalities between GBM and non-GBM with respect to race/ethnic group and sex variation were more notable than the somewhat subtle, albeit statistically significant, differences. For each subtype, the incidence rate among Blacks, Asian/Pacific Islanders, and American Indians/Alaskan Natives was substantially lower (one-fourth to one-half for GBM; about two-fifths for non-GBM) than the rate among non-Hispanic Whites, and the rate among females was about two-thirds the rate among males. These similarities suggest that within the context of a fundamental difference in the genesis of these subtypes, some aspects of the complex process of gliomagenesis are shared by these subtypes as well. The increasing calendar time trend of GBM incidence coupled with the decreasing calendar time trend of non-GBM incidence may at least partly be due to a secular trend in diagnostic fashion, as opposed to real changes in incidence of these two subtypes.

## Competing interests

The authors declare that they have no competing interests.

## Authors' contributions

RD conceived of the study, participated in the statistical analysis, and drafted the manuscript. ASD conducted the statistical analysis and provided important input into the manuscript draft. Both authors designed the study, participated in interpretation of data, and read and approved the final manuscript.

## Pre-publication history

The pre-publication history for this paper can be accessed here:

http://www.biomedcentral.com/1471-2407/11/325/prepub
